# Global research priorities related to the World Health Organization Labour Care Guide: results of a global consultation

**DOI:** 10.1186/s12978-023-01600-4

**Published:** 2023-04-07

**Authors:** World Health Organization Labour Care Guide Research Prioritization Group, Edgardo Abalos, Richard Adanu, Stine Bernitz, Lorena Binfa, Blami Dao, Soo Downe, Justus G. Hofmeyr, Caroline S. E. Homer, Vanora Hundley, Hadiza Aparajita GaladanciGogoi, Tina Lavender, David Lissauer, Pisake Lumbiganon, Robert Pattinson, Zahida Qureshi, Jeffrey S. A. Stringer, Yeshita V. Pujar, Joshua P. Vogel, Khalid Yunis, Triphonie Nkurunziza, Bremen De Mucio, Karima Gholbzouri, Anoma Jayathilaka, Adeniyi Kolade Aderoba, Veronica Pingray, Fernando Althabe, Ana Pilar Betran, Mercedes Bonet, Maurice Bucagu, Olufemi Oladapo, João Paulo Souza

**Affiliations:** UNDP/UNFPA/UNICEF/WHO/World Bank Special Programme of Research, Development and Research Training in Human Reproduction (HRP), Department of Sexual and Reproductive Health and Research, World Health Organization, Geneva, Switzerland

**Keywords:** WHO labour care guide, Research priority setting, Childbirth, Intrapartum care, Women-centred care, Childbirth experience, Evidence-based labour and childbirth care

## Abstract

**Background:**

The World Health Organization (WHO) published the WHO Labour Care Guide (LCG) in 2020 to support the implementation of its 2018 recommendations on intrapartum care. The WHO LCG promotes evidence-based labour monitoring and stimulates shared decision-making between maternity care providers and labouring women. There is a need to identify critical questions that will contribute to defining the research agenda relating to implementation of the WHO LCG.

**Methods:**

This mixed-methods prioritization exercise, adapted from the Child Health and Nutrition Research Initiative (CHNRI) and James Lind Alliance (JLA) methods, combined a metrics-based design with a qualitative, consensus-building consultation in three phases. The exercise followed the reporting guideline for priority setting of health research (REPRISE). First, 30 stakeholders were invited to submit online ideas or questions (generation of research ideas). Then, 220 stakeholders were invited to score "research avenues" (i.e., broad research ideas that could be answered through a set of research questions) against six independent and equally weighted criteria (scoring of research avenues). Finally, a technical working group (TWG) of 20 purposively selected stakeholders reviewed the scoring, and refined and ranked the research avenues (consensus-building meeting).

**Results:**

Initially, 24 stakeholders submitted 89 research ideas or questions. A list of 10 consolidated research avenues was scored by 75/220 stakeholders. During the virtual consensus-building meeting, research avenues were refined, and the top three priorities agreed upon were: (1) optimize implementation strategies of WHO LCG, (2) improve understanding of the effect of WHO LCG on maternal and perinatal outcomes, and the process and experience of labour and childbirth care, and (3) assess the effect of the WHO LCG in special situations or settings. Research avenues related to the organization of care and resource utilization ranked lowest during both the scoring and consensus-building process.

**Conclusion:**

This systematic and transparent process should encourage researchers, program implementers, and funders to support research aligned with the identified priorities related to WHO LCG. An international collaborative platform is recommended to implement prioritized research by using harmonized research tools, establishing a repository of research priorities studies, and scaling-up successful research results.

**Supplementary Information:**

The online version contains supplementary material available at 10.1186/s12978-023-01600-4.

## Background

More than one-third of maternal deaths, close to half of stillbirths and a quarter of neonatal deaths result from complications during labour and childbirth [1–3]. The majority of these deaths occur in low-resource settings and are largely preventable through timely identification of complications and interventions during labour and childbirth.

In 2018, the World Health Organization (WHO) published recommendations on intrapartum care for a positive childbirth experience [4], to ensure good-quality and evidence-based care irrespective of the setting or level of health care. These recommendations specify the evidence-based practices that should be implemented throughout labour, childbirth and the immediate postnatal period, as well as the ineffective and potentially harmful practices that should be avoided. They include new definitions of the first and second stages of labour and its duration. These recommendations mean that previous partograph designs (particularly those with alert and action lines), including the modified WHO partograph, are no longer consistent with best available evidence. Further, many partograph designs do not monitor the use of effective supportive interventions, such as labour companionship, women's posture and mobilization during labour and childbirth, encouragement of oral fluid and food intake, or the use of pain relief.

To facilitate the effective implementation of these recommendations, WHO reviewed and revised the design of the previous WHO-modified partograph. The WHO Labour Care Guide (WHO LCG) [5] was developed through expert consultations [6], iterative prototype development, and a mixed-methods evaluation in six countries [7]. The WHO LCG was designed to (1) monitor the wellbeing of women and fetuses during labour; (2) identify any deviation from normality through regular maternal and foetal assessments; (3) stimulate shared decision-making and prompt action by maternity care providers and women when deviations are identified; and (4) promote woman-centred care.

Strategies to improve the quality of intrapartum monitoring and decision-making and appropriate use of the partograph have been identified in previous WHO-led research priority setting exercises for improving maternal and perinatal health outcomes [8]. With the publication of the WHO LCG in 2020, there is a need to conduct a focused research priority setting exercise to identify key questions that will help define the research agenda in the next 5 years.

We describe a mixed-methods, multi-step approach to identify research priorities relating to the WHO LCG. This exercise is part of the WHO research roadmap to identify and support research priorities that will provide the evidence-based to effectively introduce and sustain use of the WHO LCG. The WHO research roadmap also includes coordination of research efforts, engagement with key stakeholders, including donors, and support for medium to long-term scale-up of successful strategies that will emerge from the studies conducted.

## Methods

**Fig. 1:**
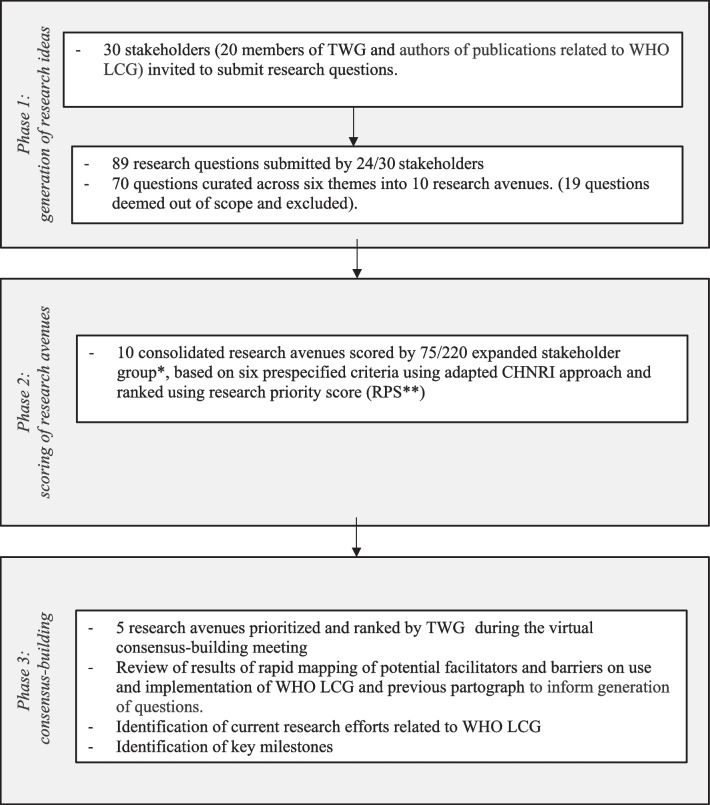
Overview of the World Health Organization Labour Care Guide (WHO LCG) research priority setting exercise. TWG: Technical Working Group; CHNRI: Child Health and Nutrition Research Initiative. * Expanded expert group includes members of the TWG (n=20), authors of publications related to WHO LCG (n=10) and additional stakeholders (members of WHO/HRP guideline development groups on maternal health, WHO HRP Alliance hub-leads, focal points of WHO collaborating centres on maternal and child health, and representatives of professional societies, women organizations, and UN agencies; n= 190); **Research priority score is based on a score of "3", "2", "1" and "0" if a participant "agrees", "neither agree nor disagrees", "disagrees" with, or is "not well informed or do not wish to score" a research question/avenue

The WHO LCG research priority setting exercise (hereafter referred to as "the exercise") used a mixed-methods, three-phased approach combining a metrics-based design with a qualitative, consensus-building exercise. The three phases (Fig. 1) were: (1) generation of research ideas or questions; (2) scoring of "research avenues" (i.e., broad ideas that could be answered through a set of research questions) using an online survey; and (3) consensus-building in a virtual stakeholder meeting. This process was developed in accordance with the 10 domains of the ﻿reporting guideline for priority setting of health research (REPRISE) [9].

### Context and scope

The exercise was set with a global perspective and focused on the population of pregnant women experiencing labour and childbirth under the care of skilled health personnel in health care facilities. It aimed to identify research priorities for improving maternal and perinatal health, experience of care and process outcomes around the time of birth using the WHO LCG, and to foster adoption and impact of this tool at scale.

To address the complex and diverse global needs related to labour and childbirth care, this exercise was set up to also consider the needs and priorities of particular subgroups of women, clinical situations, and settings. This included for example providing intrapartum care for adolescents, women in preterm labour, and women with multiple pregnancies or populations with a high burden of adverse pregnancy outcomes. Other considerations were the needs of different skilled personnel (e.g., nurses, midwives, doctors, obstetricians, etc.), the levels of health care service (primary, secondary, tertiary), and settings (e.g., low-resource or highly medicalized settings).

The intended audience for the findings of the exercise is researchers, maternity care providers, and funders, with the ultimate aim of generating research findings that can be used to design, organize, and improve labour and childbirth services. The research areas considered included clinical and health services research, and the appropriate research methods included quantitative, qualitative, and mixed methods. No specific types of research questions were pre-defined. The exercise was meant to identify research priorities in the short to medium term, i.e., research avenues that could be answered with a set of research questions within two years or less and not more than five years.

### Frameworks for research priority setting

The methodology was adapted from existing metrics-based approaches to research prioritization, including the Child Health and Nutrition Research Initiative (CHNRI) methods [10], and priority setting and agreement approaches based on the ﻿James Lind Alliance (JLA) methods [11]. Further guidance for the WHO LCG research priority setting exercise included WHO approaches to Plan, Implement, Publish and Evaluate (PIPE) research priority-setting process [12], and experience with previous WHO-led research prioritization exercises [8, 13–15].

As proposed by CHNRI, the scope of the research priorities was based on, but not limited to, the 4D framework—description, delivery, development, and discovery [10]. Within the 4D framework, this exercise was designed to identify "research avenues" that are neither too broad nor too specific, and could be answered through a set of research questions. Therefore, the output of this exercise was not formulated in the traditional PICO (population, intervention, comparator, outcome) research question format. The CHNRI method suggests that research priorities are scored using five standard criteria (answerability, effectiveness, deliverability, maximum potential impact, and equitability). This specific exercise added a sixth criterion—timeliness—to ensure that research priorities were relevant to the Sustainable Development Goals (Additional file 1: Table S1). This was followed by a virtual consensus-building meeting and electronic post-meeting exchanges of a Technical Working Group (TWG) to discuss the research priorities and determine their final ranking, what is needed to address the research priorities and key milestones.

### Governance and stakeholders

A team of WHO staff supported by external methodologists (hereinafter "the WHO team") defined the scope, developed the methodology and oversaw the conduct of the different phases of the research exercise. The WHO team has previously conducted priority-setting and consensus-driven exercises on maternal and perinatal health [8, 13, 14]. The WHO team was complemented during the consensus-building process by WHO maternal and perinatal health staff from its regional offices.

The WHO team convened a TWG of 20 purposively selected stakeholders composed of midwives, obstetricians, paediatricians, researchers, implementers and women's representatives. These individuals were from a variety of academic and research institutions, implementation organisations and non-governmental organizations, and were based in multiple geographic regions. TWG members were identified through their previous contributions to WHO normative and research activities on intrapartum care. The majority of the TWG members include the core group that developed [16] and led the testing of the WHO LCG for usability and feasibility [7]. The TWG participated in all phases: including submission of research ideas or questions, scoring "research avenues" in the online survey, and participating in the consensus-building process.

In addition to the TWG, authors of publications related to WHO LCG (n = 10) were invited to submit research ideas and questions and score research avenues. Finally, other stakeholders (n = 190) were invited to score the research avenues. These additional stakeholders were selected from a pool of members of WHO guideline development groups on maternal and perinatal health, WHO HRP Alliance hub-leads, focal points of WHO collaborating centres on maternal and child health, and representatives of professional societies, women's organizations, and UN agencies. This included obstetricians, paediatricians, midwives, and researchers from all WHO regions and service users to ensure a more comprehensive representation of relevant stakeholders with geographic representation and gender balance. By definition, these groups bring geographical and gender diversity. TWG members and other stakeholders were contacted in their individual capacity and were not reimbursed for participating in this exercise.

### Steps for identification, collection and prioritization of research avenues

The multi-phased approach for this research prioritization exercise is described in Fig. 1. The exercise was conducted between July 2021 and January 2022, from development of the scope and methodology to finalization of the consensus process.

#### Phase 1: generation of research ideas

The TWG and corresponding authors of publications related to WHO LCG were contacted and requested to submit up to five research ideas or questions related to the WHO LCG, using the online survey platform SurveyMonkey©. Given that the WHO LCG is a new tool, those selected to submit ideas or questions had to know its aims and components. Participation was anonymous and voluntary. Respondents could adopt different perspectives when submitting ideas or questions based on their background and expertise, such as frontline health workers, health managers, policymakers, educators or women's representatives. Individuals could work with their teams to develop the questions but could only make a single online submission. A set of orientation slides was shared along with the survey.

Then, two WHO team members (AKA and MBo) curated these submissions, excluded those that were out-of-scope (e.g., unrelated to labour, childbirth, and the WHO LCG), and conducted a thematic analysis of ideas and questions. Themes were developed iteratively, and research questions merged to produce "research avenues." Other members of the WHO team reviewed the research ideas and questions, the results of the thematic analysis, and the research avenues (Additional file 1: Table S2).

#### Phase 2: scoring of research avenues

An expanded group of stakeholders (including members of the TWG (n = 20), authors of publications related to WHO LCG (n = 10) and additional stakeholders (n = 190)) were invited to score the research avenues, using *a-priori* scoring criteria on SurveyMonkey©. The survey was in English. Each of the CHNRI five standard criteria and timeliness (added to this exercise) (Additional file 1: Table S1) was assigned a score of "3", "2", "1" and "0" if a participant "agrees", "neither agree nor disagrees", "disagrees" with, or is "not well informed or do not wish to score" a research avenue. Participation in this online survey was voluntary and anonymous, but general demographic and professional information was collected. Responses were not attributed to any specific person or institution.

A research priority score (RPS) was generated by summing the scores attributed to each criterion and taking an arithmetic average of the six scoring dimensions for each research avenue. No special weighting of criteria was applied. Based on the RPS, the research avenues were ranked from 1 (highest score) to 10 (lowest score), and this served as the basis for the consensus-building process (phase 3). Analysis was conducted using Microsoft® Excel for Mac Version 16.61.1 (22052000).

#### Phase 3: consensus-building

During a virtual consensus-building meeting of the TWG, consolidated results of the online scoring survey (phase 2) were shared. The exercise aimed for unanimous consensus; however, it allowed used of majority votes in cases where unanimity could not be achieved. To inform the consensus process, the WHO team conducted rapid mapping of comments on the feasibility and acceptability of WHO LCG raised while piloting the tool and since its publication. Sources used included feedback from WHO LCG user's during the usability and feasibility study [7], feedback from health workers attending WHO LCG introduction virtual sessions, published commentaries on WHO LCG up to October 2021 [17–19] and additional evidence [20]. This was complemented with a summary of existing evidence reviews on facilitators and barriers of use of the previous partograph [21–23]. The mapping helped to check the completeness of research avenues scored in phase 2, and consideration of potential gaps by the TWG during the virtual consensus-building meeting.

TWG members were asked to reflect on the survey results with considerations to re-rank, merge, reword or add new research avenues. The reflection process included verbal and written feedback (during and after the meeting). TWG members were requested to justify any change suggested to a research avenue or the survey ranking. They were also tasked with suggesting research designs to address the set priorities and to provide insights into current research efforts to address the identified priorities. This was complemented with a search (Additional file 1: Table S3) in 22 relevant registries listed in the U.S. Department of Health and Human Services (HHS), as of 8 November 2021 and updated on 17 May 2022, using the keyword "Labour Care Guide". Identified research efforts were then mapped against the defined research priorities.

## Results

Figure 1 shows an overview of the WHO LCG research priority setting exercise with each phase, described in more detail below.

### Phase 1: generation of research ideas

Of 30 stakeholders invited to submit ideas and questions, 24 individuals responded and submitted 89 research ideas and questions. Annex 2 shows how the WHO team curated the ideas and questions submitted. Nineteen ideas/questions were deemed out of scope, and the remaining 70 ideas/questions were categorized into six themes. The themes were:

- Implementation research (n = 34)
- Maternal and perinatal outcomes (n = 10)
- Women experiences (n = 9)
- Process of care—the provision of supportive care (e.g., companionship, mobility) and the use of clinical interventions (e.g., caesarean section, augmentation of labour, amniotomy, instrumental delivery, foetal monitoring, cervical dilatation monitoring) (n = 6)
- Tool development—effect of modifying thresholds in the WHO LCG alert line (e.g., lower thresholds of cervical dilatation and time limits of labour progress) (n = 6)
- Organization of labour and childbirth care (e.g., referrals, teamwork, patient flow, human and physical resource requirements, and shared decision-making between a woman and her maternity care provider) (n = 5).

In the first iteration, similar ideas/questions were consolidated into 40 groups. After that, the initial attempt to frame research avenues yielded 26 research topics. These were consolidated into ten research avenues for scoring by the expanded group of stakeholders.

### Phase 2: scoring of research avenues

Seventy-five out of 220 stakeholders (34%) who received the online scoring survey responded. Respondents were from a variety of professional backgrounds, affiliations, and geographies (Additional file 1: Fig. S4). Most respondents were researchers and obstetricians, working in research institutions, universities or public hospitals. The Eastern Mediterranean regions was under-represented.

Table 1 shows the average scores and ranking of the ten research avenues by all participants. Research avenues related to implementation research, process of care, women's experiences, and maternal and perinatal outcomes were ranked top-4. Research avenues related to the organization of care and resource utilization ranked lowest in the scoring survey. Detailed sub-group analysis by score domain and respondents (TWG and all respondents) are presented in Additional file 1: Tables S5.A and S5.B. The same research avenues were ranked as top-4 priorities across TWG and all respondents.

**Table 1:** Ranking of 10 research avenues related to World Health Organization Labour Care Guide (WHO LCG): results of the online scoring survey (phase 2)

Initial ranking	Initial theme	Initial research avenue	Average score*
1	Implementation research	What are the facilitators and barriers to implementing the WHO LCG with high fidelity among different cadres, levels of care and settings?	2.63
2	Process of care	What is the effect of the WHO LCG on the provision of supportive care (e.g., companionship, mobility) and the use of clinical interventions (e.g., caesarean section, augmentation of labour, amniotomy, instrumental delivery, foetal monitoring, cervical dilatation monitoring) during labour and childbirth?	2.61
3	Women experiences	What is the effect of the WHO LCG on the experience of care (e.g., satisfaction, respectful maternity care) during labour and childbirth?	2.59
4	Maternal and perinatal outcome	What is the effect of the WHO LCG on short- and long-term maternal and perinatal health outcomes?	2.54
5	Implementation research	What are the most effective approaches (e.g., education and training, monitoring and feedback, digital LCG, ownership of the WHO LCG and presentation in a health facility by the woman) to implement WHO LCG with high fidelity among different cadres, levels of care and settings?	2.52
6	Implementation research	What are the most effective education and training approaches on the WHO LCG to improve knowledge, attitudes, and intrapartum care practices of different cadres?	2.49
7	Tool development	Is the use of the WHO LCG to monitor labour progress feasible, safe and effective in improving outcomes in special situations (e.g., induction of labour, breech presentation, premature labour, epidural analgesia, twin pregnancy)?	2.48
8	Organisation of care and resource utilisation	What is the effect of the WHO LCG on the organisation of labour and childbirth care (e.g., referrals, teamwork, patient flow, human and physical resource requirements, shared decision making between the woman and maternity care provider)?	2.37
9	Tool development	What is the effect of modifying thresholds in the WHO LCG alert line (e.g., lower thresholds of cervical dilatation and time limits of labour progress, threshold for fetal descent) on maternal and perinatal outcomes, use of clinical interventions and organisation of care, in various levels of care and different settings?	2.37
10	Organisation of care and resource utilisation	How cost-effective is the WHO LCG in various levels of care and different settings?	2.32

^*^Highest possible score – 3.00 (highest priority). Based on online scoring by 75 experts

### Phase 3: consensus-building

During the virtual meeting, the TWG (17 out of the 20 members attended) reviewed the results of the online scoring survey (phase 2) and unanimously agreed to merge some of the 10 research avenues and modify the rankings after discussions. WHO headquarters (6) and regional staff (4), and methodologist (2) facilitated the process. The process was informed by considerations of the feasibility and acceptability (Additional file 1 Table S6) of WHO LCG, based on the rapid mapping of the evidence and ongoing research.

The 10 original research avenues were thus revised and merged into five research avenues (Additional file 1 Table S7). The top-3 research avenues agreed upon by TWG include (1) optimizing implementation strategies of WHO LCG; (2) improve understanding of the effect of WHO LCG on maternal and perinatal outcomes, experience of care and process outcomes during labour and childbirth; and (3) assess the effect of the WHO LCG in special situations or particular settings. Additional file 1 Table S7 summarizes the results of the consensus-building process and justifications for merging the 10 research avenues into five and their ranking.

Table 2 describes why each research avenue prioritized is important, the knowledge gaps, and what is needed to address these gaps. To optimise implementation strategies of the WHO LCG, studies are needed to improve understanding of barriers and facilitators, effects on health providers’ knowledge, attitudes and skills, and optimal approaches to engage with labouring women. Rigorous monitoring and evaluation of "real-life" WHO LCG implementation in health facilities and at the sub-national or national levels is also required. Multi-country, multi-phased studies, in a variety of levels of care and settings would be preferred to evaluate the effect of introducing WHO LCG on health, experience and process outcomes. Lastly, assessing the effect of the WHO LCG in special situations or particular settings may require the design and evaluation of an optimised tool and implementation strategy to maximise the effects of WHO LCG. A range of studies using multiple methodological approaches would be required to address the research priorities, including trial designs, observational, qualitative and modelling studies.

**Table 2:** Consensus-based research priorities (phase 3) related to World Health Organization Labour Care Guide (WHO LCG)

Consensus-based theme	Consensus-based research avenue	Why?	What is needed?
1. Optimise implementation strategies of WHO LCG	What are the most effective approaches* to implement WHO LCG with high fidelity among different cadres, levels of care and settings? *e.g., education and training, monitoring and feedback, digital LCG, ownership of the WHO LCG, and presentation in a health facility by the woman	• To optimise strategies to introduce and sustain implementation of the WHO LCG • To improve understanding of facilitators and barriers, to develop implementation strategies to optimise introduction, scale-up and sustainability of the WHO LCG, in different settings and among different cadres of health workers	• Studies to assess barriers and facilitators for introducing and using WHO LCG, acceptability, factors affecting use and compliance, health workers' attitudes, education and training needs, behaviour changes and women's experience • Design and evaluate WHO LCG implementation strategies focusing on achieving adoption and high fidelity with WHO LCG use and improvement of process outcomes, in different settings • Compare different WHO LCG implementation strategies, focusing on achieving optimal adoption and high-fidelity use of the WHO LCG and improving process outcomes • Understand the effect of the use of WHO LCG on knowledge, attitudes, and intrapartum care practices among different health cadres • Understand approaches to engage with women and communicate about the importance of respectful labour monitoring • Consideration should be given to multi-phased studies, in a variety of levels of care and settings, including: – Pre-implementation phase: introductory pilot in a small sample of women or health facilities or both – Formative phase to co-develop the accompanying implementation strategies • Rigorous monitoring and evaluation of "real-life" WHO LCG implementation in health facilities and at the sub-national or national levels • Proposed research designs include: – Quantitative (e.g., surveys, before-after, interrupted time series) – Qualitative studies – Mixed-methods studies
2. Improve understanding of the effect of WHO LCG on outcomes	What is the effect of the WHO LCG on maternal and perinatal health outcomes, and the process and experience of labour and childbirth care*? * short-term maternal and perinatal health, provision of care/use of interventions, experience of care	••••To increase understanding of the effectiveness of WHO LCG on providing evidence-based intrapartum care, reducing unnecessary interventions, and improving maternal and perinatal health, experience of care and process outcomes	• Evaluate the effectiveness of introducing WHO LCG on maternal and perinatal health outcomes, process outcomes, and experience of care in different settings • Multi-country, multi-phased studies, in a variety of levels of care and settings, including: – Formative phase to co-develop the accompanying implementation strategies to optimise effects of WHO LCG; especially the development of a context-specific implementation strategy, including complex interventions – Implementation and evaluation of WHO LCG implementation strategy targeting labour and childbirth processes or outcomes or both • Proposed research designs include: – Stepped wedge trials – Participant-action-research cycles with women and health workers – Observational studies: before-after, interrupted time series – Modelling studies
3. Assess the effect of the WHO LCG in special situations or particular settings	Is the use of the WHO LCG to monitor labour progress feasible, safe and effective in improving outcomes in special situations* or particular settings**? * e.g., induction of labour, breech presentation, premature labour, epidural analgesia, twin pregnancy, previous caesarean section **e.g., limited or no access to caesarean section, remote areas, low-resource or highly medicalized settings	• To increase understanding of the feasibility, safety and effectiveness of WHO LCG in special situations or particular settings where referral to a higher level of care may be required	• Evaluate safety, efficacy, and effectiveness of introducing WHO LCG on maternal and perinatal health outcomes, process outcomes, and experience of care in special situations or particular settings. This may require the design and evaluation of an optimised tool and implementation strategy focused on addressing the issues related to the special situations or particular settings to maximise the effects of WHO LCG • Understand unintended consequences of the introduction and use of WHO LCG in special situations or particular settings • Efficacy studies comparing different alert thresholds for the management of special situations or in particular settings • Proposed research designs include: – Randomised controlled trials – Modelling studies
4. Understand effects of WHO LCG on organisation of care and resource requirements	What is the effect of the WHO LCG on the organisation of labour and childbirth care* in various levels of care and different settings? * e.g., referrals, teamwork, patient flow, human and physical resource requirements, shared decision making between the woman and maternity care provider	• To optimise allocation of resources and organisation of labour and childbirth care	• Design and evaluate strategies to optimise organisation of care and allocation of resources during labour and childbirth care • Understand unintended consequences of the introduction and use of WHO LCG in the organisation of care (e.g., overcrowding of labour ward due to longer labours) • Studies to assess barriers and facilitators, acceptability, health workers' attitudes, behaviour changes and women's experience • Proposed research designs include: – Quantitative (e.g., surveys, before-after, interrupted time series) – Qualitative – Mixed-methods studies
5. Assess the economic impacts of the W.HO LCG	How cost-effective is the WHO LCG in various levels of care and different settings?	• To inform decision-making about implementing strategies for introducing and sustaining the use of WHO LCG	• Understand the economic impact of introducing and using WHO LCG • Understand the economic impact of different strategies introducing and using WHO LCG • Cost estimates of introducing and sustaining the use of WHO LCG, and its implementation strategies • Proposed research designs include: – Resource implications, cost evaluations and cost-effectiveness studies embedded in studies on the effects of WHO LCG on outcomes, including evaluation of implementation strategies – Cost evaluation modelling

Nine ongoing or planned research efforts were identified (Additional file 1: Table S8), mainly responding to the second priority to improve understanding of the effect of WHO LCG on maternal and perinatal outcomes, and the process and experience of labour and childbirth care [24–28]. One completed, but noy yet published study in India was identified under the theme “optimize implementation”. Eight studies were identified under the theme “improve understanding of effect of WHO LCG on outcomes”, three in Europe (England, Norway, Sweden), three in Asia (India), and two in Africa (Botswana, Zambia, Zimbabwe). Of those, only two Indian studies have completed recruitment, and the study in Botswana is ongoing. None covered the theme “Assess the effect of the WHO LCG in special situations or particular settings (Additional file 1: Table S8). Key milestones across the different priorities include setting up a platform to maximize research network collaboration, a repository of research efforts, harmonization of research tools and outcomes, and evidence synthesis to facilitate sharing experience.

## Discussion

### Main findings

A diverse group of stakeholders agreed that the most pressing research priorities related to the WHO LCG during the SDG era are research to (1) optimize implementation strategies, (2) improve understanding of the effect of WHO LCG on maternal and perinatal outcomes, experience of care, and process outcomes during labour and childbirth care, and (3) assess the effect of the WHO LCG in special situations or settings. The lower-ranked priorities were research to (4) understand the effects of WHO LCG on the organization of care and resource requirements, and (5) assess the economic impacts of the WHO LCG. There was agreement between the scoring by the larger group of stakeholders using an online survey and the ranking by the experts in TWG during the consensus-building meeting on the highest and lowest groups of research priorities.

## Interpretation

The ranking of optimizing WHO LCG implementation strategies as the highest research priority acknowledges that implementing the tool is critical to providing quality intrapartum care, including both evidence-based process and experience of care, to ultimately improve maternal and perinatal outcomes. It is expected that better monitoring of labour and childbirth with WHO LCG will improve maternal and perinatal outcomes and experiences. However, a better understanding of successful implementation approaches in different contexts (cadres, levels of care, and settings) is needed. Lessons learned from the implementation of older partograph designs (e.g., need for supervision and training) could inform WHO LCG implementation strategies [22].

Giving priority to implementation research seems in line with efforts to improve implementation strategies and sustainability of evidence-based interventions globally [29, 30]. Implementing the WHO LCG should be accompanied by effective strategies to maximize adherence, not only with using the tool but by ensuring clinical decision-making is evidence-based. Research studies considering implementation strategies to introduce and implement the WHO LCG must be relevant to local needs and realities (e.g., co-developed interventions after the formative research phase, protocols and interventions adapted to local context). In some settings, these implementation strategies will need to be accompanied by additional activities to implement key components of the WHO intrapartum care model (e.g., companion of choice, mobility), improve management (e.g., intrapartum care protocols, optimize referral systems) [31] and train maternity care providers. The available national and health facility resources and policy contexts, and how they can be optimised, will also need to be explored within WHO LCG implementation research efforts. Any such implementation research should include rigorous monitoring and evaluation approaches, including assessment of potential unintended consequences (e.g., adverse events). Indeed, monitoring and audit of practices, including correct completion, decision-making, referrals, and maternal and perinatal outcomes, has been already described for implementation of the previous partograph [22].

The second research priority calls for designing context-specific approaches to better understand the effect of WHO LCG on pregnancy outcomes in different settings. This includes: (1) how WHO LCG affects the provision of supportive care (e.g., companionship, mobility) and the use of clinical interventions (e.g., caesarean section, augmentation of labour, amniotomy, instrumental delivery, foetal monitoring, cervical dilatation monitoring) during labour and childbirth; or (2) What is the effect of the WHO LCG on short- and long-term maternal and perinatal health outcomes? This priority recognizes that strong evidence on the effectiveness of WHO LCG on maternal and perinatal outcomes, and the process and experience of labour and childbirth care will potentially support adoption, increase acceptability, and use. It is noteworthy that trials of earlier partograph designs has shown limited effects on these outcomes, and that these effects probably depend on adherence to clinical management protocols rather than using a tool alone [21–23].

Priority was given to assessing the WHO LCG in its current form over proposing adaptations for special situations or settings. Therefore, the third priority recognizes the importance of robust evidence generation to inform adaptations of the tool (e.g., modifications of thresholds in the WHO LCG in settings with limited access to caesarean section), or changes in its implementation strategies in special situations or particular settings. For example, (1) those with limited or no access to caesarean section and higher-level care, (2) settings with high rates of overmedicalized labour care (e.g., high frequency of augmentation, caesarean section), or (3) specific clinical situations (e.g., induction of labour, epidural, vaginal births for breech presentation, or women with previous caesarean section). It was noted during the consensus-building meeting that, for a given context, high research priorities might differ depending on: levels of acceptability of WHO LCG (high vs. low), frequency of special situations (e.g., high vs. low rates of induction of labour, breech presentation, premature labour, epidural analgesia, twin pregnancy, previous caesarean section) or resource level (e.g., limited or no access to caesarean section, remote areas, lower level health facilities).

Although closely related with optimizing WHO LCG implementation strategies, assessment of the economic impacts of the tool, resource utilization, and organization of care were identified as research areas warranting investigation. Indeed, in their mixed-methods study, Vogel et al. noted that staff training, workload organization, and enabling policies guaranteeing accessible standard protocols, equipment, and medical supplies, and restructuring labour suites are necessary to effectively implement the WHO LCG in different contexts [7].

The research avenues provided in this paper are framed broadly, and researchers will need to develop more refined questions for investigation. They will also need to identify the correct research methods and study designs to address these questions [10], based on the methodologies proposed in this exercise. It is evident that multiple studies using diverse methodological approaches are needed to address all research priorities. The ongoing and planned research studies described in Additional file 1: Table S8 provide examples of a research questions and methodological approaches that could be applied or adapted for future studies. Ideally, these studies will be conducted in a variety of settings (urban/rural, low/high resource, settings with minimal interventions/highly medicalized environments, uncomplicated labour and birth/emergencies), levels of care (primary, secondary, tertiary) and among different cadres of skilled personnel (e.g., nurses, midwives, doctors, obstetricians). However, a single research activity could simultaneously address different research avenues. For example, a single hybrid implementation-effectiveness study can address sub-questions using quantitative, qualitative and economic evaluation.

Data collection tools and implementation measures including training protocols, should be harmonised, to facilitate comparisons across studies, and to enable evidence synthesis. As part of its core functions, WHO can raise partnerships, networks and alliances, and initiatives to give more credence to WHO LCG research priorities including optimizing its implementation. The WHO research roadmap on WHO LCG will encompass efforts to disseminate the results of the present prioritization exercise to the global maternal and perinatal health research community and other stakeholders, including research funders, and track research studies responding to the identified research priorities. The findings will be disseminated using peer review publications, meetings with stakeholders, and WHO online platforms. An online platform under WHO's managerial control can coordinate collaboration with multiple stakeholders, call for funding, provide accountability and the track progress of research priorities.

### Strengths and limitations

This exercise utilized a rigorous process based on up-to-date guidance [9, 12]. This incorporated a multi-phased process, combining metrics-based [10, 32], and qualitative, consensus-building workshop approaches [11]. The process included the collection, analysis, and ranking of research priorities transparently from a diverse group of relevant stakeholders. Its methods are replicable [33]. In line with the CHNRI method [10, 32], the initial ideas/questions for this exercise were generated using an online survey platform, thus minimizing the influence that people submitting research questions have on one another. Since the WHO LCG is a new tool, it is unlikely that any research priority emanating from this exercise would already have sufficient existing evidence. Nonetheless, we corroborated the priorities identified with emerging evidence on the feasibility, acceptability, and usability of the WHO LCG [7, 20], and opinions in three recent commentaries [17–19].

We reported both the highly ranked and lowest ranked research. As previously observed in research prioritization exercises, comprehensive coverage of all research ideas is challenging [34]. We mitigated participating stakeholders' biases by gathering research ideas/questions from a diverse group of stakeholders representing different cadres groups and geographies. Despite our effort to achieve a reasonable balance in occupation, gender, and geographical spread, we observed a relative over-representation of obstetricians and researchers over other cadres in the online scoring survey. We think that any potential bias in the raking of questions introduced by the over-representation of obstetricians and researchers in phase 2 would have been redressed by the TWG, a more balanced group, during the consensus-building meeting. We also noted a relatively low response rate (34.1%) in the scoring survey, similar to other global research prioritization efforts [8, 14, 15].

## Conclusion

Optimizing implementation strategies and improving understanding of the effects of WHO LCG on maternal and perinatal outcomes, and the process and experience of care during labour and childbirth are the top research priorities before the end of the SDG era. The systematic and transparent process employed in this research prioritization exercise should encourage researchers, policymakers, and funders to support the conduct of WHO LCG research that is aligned with the identified priorities. This exercise showed that a range of studies using multiple methodological approaches is needed to address the research priorities. Going forward an international collaborative platform could maximise efforts to implement research studies addressing prioritized research avenues, using harmonized research tools, backed up with a repository of studies responding to identified research priorities, and with rapid scale up of successful research results.

## Supplementary Information

**Additional file 1: Table S1.** Criteria for scoring research questions during the online survey (phase 2) - based on Child Health and Nutrition Research Initiative (CHNRI) methodology. **Table S2.** Process of curating research questions (phase 1) relating to WHO LCG . **Table S3.** Results of focused searches of research efforts. **Fig. S4.** Profile of respondents to the WHO LCG scoring online survey (phase 2). **Table S5A.** Results of scoring research priorities relating to WHO LCG: total and by domain. **Table S5B**. Results of scoring research priorities relating to WHO LCG: all respondents and TWG. **Table S6.** Rapid mapping of feasibility and acceptability of WHO Labour Care Guide. **Table S7.** Ranking of research priorities related to WHO LCG: results of the consensus-building process* (phase 3). **Table S8.** Ongoing research covering research priorities related to WHO LCG and key milestones.

## Data Availability

All data generated and analysed are available in the manuscript and Additional file 1.

## Abbreviations

CHNRI: Child Health and Nutrition Research Initiative
JLA: James Lind Alliance
LCG: Labour Care Guide
REPRISE: Reporting guidelines for priority setting of health research
SDG: Sustainable Development Goals
TWG: Technical working group
WHO: World Health Organization
