# Supplementary material for: Global research priorities related to the World Health Organization Labour Care Guide: results of a global consultation
Source: Reprod Health. 2023 Apr 7;20:57. doi: 10.1186/s12978-023-01600-4 (PMC10082494; doi:10.1186/s12978-023-01600-4)
Supplement: Supplementary file 1 — Additional file 1: Table S1. Criteria for scoring research questions during the online survey (phase 2) - based on Child Health and Nutrition Research Initiative (CHNRI) methodology. Table S2. Process of curating research questions (phase 1) relating to WHO LCG . Table S3. Results of focused searches of research efforts. Fig. S4. Profile of respondents to the WHO LCG scoring online survey (phase 2). Table S5A. Results of scoring research priorities relating to WHO LCG: total and by domain. Table S5B. Results of scoring research priorities relating to WHO LCG: all respondents and TWG. Table S6. Rapid mapping of feasibility and acceptability of WHO Labour Care Guide. Table S7. Ranking of research priorities related to WHO LCG: results of the consensus-building process* (phase 3). Table S8. Ongoing research covering research priorities related to WHO LCG and key milestones. [file 12978_2023_1600_MOESM1_ESM.docx]

**Table S1: Criteria for scoring research questions during the online survey (phase 2) - based on Child Health and Nutrition Research Initiative (CHNRI) methodology**

| **Criteria** | **Definition** |
| --- | --- |
| Answerability | likely to be answered through ethical research |
| Effectiveness | potential to improve outcomes (e.g., reduce maternal and/or newborn morbidity or mortality, improve the experience of care) |
| Deliverability | potential to lead to solutions that are deliverable through existing health systems/contexts, including low resource settings |
| Maximum potential impact | potential to produce scalable results that are likely to have a large effect on maternal and neonatal health and well-being |
| Equitability | potential to lead to interventions that will preferably be available and accessible to the entire population irrespective of income or socio-economic strata or to high-interest populations |
| Timeliness | potential to complete the research in the short- to medium-term. Preferably within two years or less and not more than five years |

**Table S2. Process of curating research questions (phase 1) relating to WHO LCG**

| Themes | All submitted research ideas and questions | Out of scope ideas/questions were removed and similar ideas/questions consolidated | Framing research avenues - first draft | Research avenues |
| --- | --- | --- | --- | --- |
| Implementation Research | 1. To explore what is achievable, acceptable, and applicable clinical guidelines to accompany the LCG (i.e., clinical practice guidelines indicating what to do when LCG thresholds are met) in settings with different resource levels (notably, the WHO 2018 guidelines are too unspecific to work as such decision-support for what to do when thresholds are met). Population: Health providers from different clinical settings Exposure: Co-creation workshops, structured around the WHO-integrate evidence to decision framework Design: Descriptive study Outcome: Co-created achievable, acceptable, and applicable clinical guidelines for what to do when LCG thresholds are met, including simple tools for how to adapt to different clinical settings with different levels of resources as well as implementation plans (notably, such approach is urgently called for: Improving the quality of WHO guidelines over the last decade: progress and challenges - The Lancet Global Health; WHO \| Enhancing WHO's standard guideline development methods; The injustice of unfit clinical practice guidelines in low-resource realities - The Lancet Global Health; Beyond too little, too late and too much, too soon: a pathway towards evidence-based, respectful maternity care worldwide (healthynewbornnetwork.org)  2. Assess the potential challenges to overcome in implementing WHO LCG in low- and middle-income countries: qualitative with health staff (healthcare providers) as a multicentre study. Even this can be a mixed-methods study.  3. What are the most efficient ways of educating healthcare providers on LCG use and adaptation? This can be done as a post-intervention survey. Healthcare providers can be surveyed on the practical, effective ways of getting attention and knowledge on LCG. The LCG Manual will not be read by everyone in the facilities. Need their opinion about the effective practical ways.  4. Level of implementation of WHO LCG in several low- and middle-income countries. Can be done using the post-implementation quantitative (including the satisfactory adherence rate to LCG) and qualitative (health staff on training, challenges, barriers, and enthusiasm) methods. Can compare maternal and perinatal outcomes that occurred in the latent phase 5. What are the facilitators and barriers to effective use of the WHO Labour Care at the different health care levels and how can they be harnessed or addressed?  6. Do healthcare workers who are trained to use the WHO Labour Care Guide find it more helpful in their labour-management than those who use other existing tools after adequate training?  7. What is the optimum training schedule that would ensure continued correct use of the WHO Labour Care Guide at the different health care levels?  8. What are the views and experiences of staff who are using the labour care guide, compared to those who are not using it (RCT or before and after)  9. What are the characteristics and mechanisms of effect in 'high performing' sites that have implemented the LCG with high fidelity and good clinical and psychological outcomes for service users and staff?  10. How can the mechanisms of effect in high-performing sites be transferred to lower-performing sites? 11. What is the most effective way to implement LCG in routine care in resource-limited settings, and what are the effects of LCG implementation on the process of care, satisfaction and health outcomes? Particular attention to obstetrician-led vs. midwifery-led care settings; settings with or without national RMC policies / supportive policy frameworks; settings with higher versus lower obstetric intervention (caesarean section, induction of labour, Augmentation) rates  12. Implementation research: identifying effective approaches to national or facility-level policy changes to support/promote/encourage LCG use / supportive care interventions.  13. Given the LCG complexity, reminders, and need for timely assessments, a digital LCG is a high priority. Translating the LCG into digital format to encourage adherence/use reminders is a key question.  14. What are the most effective training strategies for health workers using LCG? Consideration of: different cadres; different languages; different contexts; use of novel/remote/online training approaches  15. What are the perceptions of staff who have adopted the use of the LCG?  16. What contextual factors influence its acceptability.  17. What individual and contextual (facility and wider setting) factors influence adoption and correct use of the LCG - how can this knowledge be used to improve uptake and usage.  18. Would the implementation of scheduled training and regular certification and recertification, at defined intervals of all health care workers involved in labour and newborn care at peripheral/rural health facilities; tied to an accreditation program of the health care facility itself, by an international body, lead to improvement and sustainability of good maternal and newborn outcomes? Suppose there is a breakthrough improvement in labour care and the outcome of pregnant mothers and their newborns. In that case, there is a need for every country at the facility level to map the existing/current level of care and identify deficiencies. This should be followed by the implementation of an integrated program that addresses all health care gaps. The program needs to be updated and renewed at regular intervals. Finally, measurement of outcomes after implementation will assess success and need for modification and /or upgrading the program—a before and after mixed-method research activity. At a designated number of facilities for six months, the current partograph would continue to be used - in the research activity to include FGD's with facility staff and mothers. Then introduce the LCG with appropriate training - evaluate after six months of use - evaluate set of maternal and newborn outcomes and the same set of FGD's. Implementation science study design -in a group of selected facilities - public, private, and faith-based introduce the LCG with appropriate training and evaluate maternal and newborn outcomes after 3-6 months. This will also provide an insight into the acceptability and usability of the tool in relation to the number of staff available at the site- as the ratio of patient to a practitioner is different in private, faith-based, and public. To start country activities with a stakeholder meeting to inform them of this tool in detail, get their buy-in, then introduce the research activities  19. What education and change strategies are required to ensure that midwives, obstetricians, and women understand the different phases of labour? Consideration balance between gatekeeping & unnecessary admission/intervention - education both qualified professional and future students. 20. What education and change strategies are required to ensure the LCG is implemented as a woman-centred approach to care rather than simply a monitoring tool?  21. To evaluate the effectiveness and feasibility of using LCG in PHCs for improved labour outcome". 22. To explore the acceptability and determinants (barriers and facilitators) of LCG implementation in a low resource setting."  23. To identify the best implementation strategies and service delivery models to facilitate uptake of LCG by healthcare providers."  24. Assess the obstetric (midwifery) team competencies as a strategy to deliver the WHO LCG in medicalized healthcare facilities  25. Identify health workers perceived barriers to adopting the WHO LCG as an input to promote its implementation in medicalized healthcare facilities  26. Identify childbearing women's experience when receiving care based on the WHO LCG as an input to foster its implementation.  27. Identify stakeholders perceived barriers to adopting the WHO LCG as a strategy to promote its implementation in countries where it is not implemented  28. What are the operational barriers to implementing the LCG with high fidelity at various levels of care (primary, secondary, tertiary), and what strategies can be employed to overcome these barriers? 29. To evaluate healthcare workers' experiences of facilitators and barriers when implementing the WHO LCG in high resource settings.  30. Evaluation of the effects of different training approaches on the utilization of the labour care guide in low resources settings  31. Evaluate health worker training as a strategy to deliver the WHO LCG in healthcare facilities in low resource settings  32. How can midwives be best supported to use the LCG in low-resource settings? 33. Can the LCG be implemented in a way that ensures the woman owns it and brings it to the healthcare provider? This would empower her and provide an understanding of phases of labour 34. Is a mobile version of the labour care guide feasible and acceptable to healthcare providers in low-resource settings? | 1. What are the optimal strategies to implement the WHO LCG in various levels of care and settings? Particular attention to primary vs. secondary vs. tertiary care, obstetrician-led vs. midwifery-led care settings; settings with or without national RMC policies / supportive policy frameworks; settings with higher versus lower obstetric intervention (caesarean section, induction of labour, Augmentation) rates, HIC vs. MIC vs. LIC,  2. What are the facilitators and barriers to implementing the WHO LCG (or its sections) with high fidelity at various levels of care (primary, secondary, tertiary) and in different settings (LMIC vs. HIC, national vs. facility-level factors)?  3. How do health worker characteristics and perceptions influence the acceptability and uptake of the WHO LCG? 4. How do the perspectives of different stakeholders (e.g., health workers, managers, policymakers) influence the acceptability and implementation of the WHO LCG?  5. What are the views and experiences on intrapartum care of skilled care personnel using the labour care guide compared to those not using it? (RCT or before and after)  6. What are the characteristics and mechanisms of effect in 'high performing' sites that have implemented the LCG with high fidelity and good clinical and psychological outcomes for service users and staff?  7. How can the LCG mechanisms of effect in high-performing sites be transferred to lower-performing sites?  8. What contextual factors influence LCG acceptability and implementation (high fidelity?) at various levels of care in different settings?  9. Is it feasible, safe, and efficacious for pregnant women to own the WHO LCG and present it for intrapartum care and referrals? 10. Is a digital version of the WHO LCG feasible and acceptable to different cadres in different levels of care and settings? 11. Is training on intrapartum care competencies facilitated by using WHO LCG among different cadres of relevant health workers (e.g., nurses, midwives, doctors, obstetricians-gynaecologists, etc.)? Different settings?  12. What is the optimal training and recertification period for healthcare providers on the use of WHO LCG that maintain provider competencies in intrapartum care or its elements (such as supportive care, pain relief in labour, cervical assessment, intrapartum fetal monitoring, the decision for labour interventions and referrals)? Consideration of different cadres; different languages; different contexts; use of novel/remote/online training approaches 13. What is the optimal training and recertification period on intrapartum care competencies and use of WHO LCG among different cadres of relevant health workers (e.g., nurses, midwives, doctors, obgyns, etc.)? Different settings?  14. What is the impact of different training approaches on utilizing the WHO LCG in various settings (HIC, MIC LIC)?  15. What is the impact of different training approaches on the utilization of the WHO LCG in various levels of care (primary, secondary and tertiary) or cadres of care?  16. What is the impact of different training approaches on the WHO labour care guide to facilitate a woman-centred approach? 17. Does training on the WHO LCG or its sections improve competencies in intrapartum care or its elements (such as supportive care, pain relief in labour, cervical assessment, intrapartum fetal monitoring, the decision for labour interventions, and referrals) among different cadres of relevant health workers (e.g., nurses, midwives, doctors, obgyns, etc.)? | 1. What are the most effective approaches for training on the utilization of the WHO LCG in various levels of care and different settings? 2. What is the impact of training on the utilization of WHO LCG in intrapartum care knowledge, attitudes, and practices among different cadres? 3. What are the most effective strategies to implement the WHO LCG in various levels of care and different settings? 4. Is a digital version of the WHO LCG feasible and acceptable to other staff cadres/ in various levels of care and settings? 5. What are the facilitators and barriers to implementing WHO LCG with high fidelity at various levels of care and in different settings?  6. What individual and contextual factors influence the acceptability and usability of the WHO LCG at various levels of care and in different settings?  7. Is it feasible, safe, and efficacious for pregnant women to own the WHO LCG and present it for intrapartum care and referrals? 8. How do views and experiences of intrapartum care vary between skilled care personnel who are using the WHO LCG compared to those who are not using the WHO LCG? | 1. What are the most effective approaches (e.g., education and training, monitoring and feedback, digital LCG, ownership of the WHO LCG, and presentation in a health facility by the woman) to implement WHO LCG with high fidelity among different cadres, levels of care and settings? 2. What are the most effective education and training approaches on the WHO LCG to improve knowledge, attitudes, and intrapartum care practices of different cadres? 3. What are the facilitators and barriers to implementing the WHO LCG with high fidelity among different cadres, levels of care, and settings? |
| Organization of care and resource utilization | 35. Does the use of the labour care guide improve teamwork among different types of cadres in low resources settings? Ensure that research is not limited to English speaking countries only 36. Are there any settings where LCG adoption is associated with unintended adverse consequences?  37. What impact on patient flow, hospital systems, and human and physical resource requirements does adoption of the LCG have in practice (e.g., changing definition of active labour may alter requirements in the antenatal ward, and allowing longer in labour may influence labour ward congestion, but perhaps reduce theatre utilization)? 38. What impact does the LCG have on resource utilization compared to the modified partograph? 39. Does the use of the labour care guide improve the quality of referrals of labouring women from peripheral health centres? | 18. What is the effect of the WHO LCG on the decision-making process for intrapartum care, including provision/access to supportive care? 19. Does the use of the WHO LCG improve the quality of referrals/information sharing during referrals/takeover of labouring women from peripheral health centres? 20. Does the use of the WHO LCG improve teamwork among different cadres of intrapartum care providers in various levels of care and settings? 21. How cost-effective is the use of WHO LCG (+ recommendation) in managing women in labour compared with usual intrapartum care without WHO LCG? 22. How does the use of WHO LCG (+ recommendation) affect patient flow and human and physical resource requirements compared with usual care? | 9. What is the impact of the introduction of the WHO LCG on the shared decision-making process for intrapartum care between a woman and a skilled attendant? 10. What is the impact of the introduction of the WHO LCG on the quality and timeliness of referrals of labouring women from peripheral health centres? 11. What is the impact of the introduction of the WHO LCG on information sharing during referrals/take over of labouring women? 12. What is the impact of the introduction of the WHO LCG on teamwork among different cadres of intrapartum care providers in various levels of care and other settings? 13. Which are the most cost-effective strategies to introduce the WHO LCG with a co-designed implementation strategy in various levels of care and different settings? 14. What is the effect of the introduction of the WHO LCG on patient flow and human and physical resource requirements in various levels of care and different settings? | 4. What is the effect of the WHO LCG on the organization of labour and childbirth care (e.g., referrals, teamwork, patient flow, human and physical resource requirements, shared decision making between the woman and care provider)? 5. How cost-effective is the WHO LCG in various levels of care and different settings? |
| Process of care | 40. Use of labour care guide with infusion pumps if oxytocin was indicated and graphic Doppler Fetal monitoring VS current care. Outcomes; reduction of caesarean section; reduction of babies with HIE and 5 min Apgar score <7. 41. Does the use of the labour care guide a) reduce labour interventions, B) reduce maternal and neonatal morbidity/mortality outcomes?  42. To determine the effect of the use of LCG on Caesarean section rate for low-risk women in low resource settings 43. To investigate the effect of the WHO LCG compared to the WHO partograph on maternal and neonatal outcomes and obstetric interventions through a multicentre cluster randomized strategy trial in high resource settings 44. Does using the LCG increase women's access to the supportive care elements - especially companionship and mobility?  45. What impact does the LCG have on the use of unnecessary labour interventions compared to other labour monitoring tools in all settings [HIC, MIC, LIC]? | 23. Does the introduction of WHO LCG increase the use of supportive care elements of intrapartum care, such as companionship and mobility? 24. What are the effects of LCG implementation on the process of care, e.g., intrapartum fetal surveillance, labour progress monitoring? 25. What is the effect of the WHO LCG compared to other labour monitoring tools on the use of intrapartum clinical interventions, such as….? 26. What is the effect of the WHO LCG compared to other labour monitoring tools on the use of unnecessary labour interventions in different levels of care [primary, secondary, and tertiary] and settings [HIC, MIC, LIC]? 27. What effect does the use of/the introduction of the WHO LCG have on cesarean section rate in different settings (LIC, MIC, and HIC)? | 15. What is the impact of the introduction of the WHO LCG on the use of clinical interventions during labour and childbirth (e.g., cesarean section, augmentation of labour, amniotomy, instrumental delivery)? 16. What is the impact of the introduction of the WHO LCG on the uptake of supportive care elements of intrapartum care, such as companionship and mobility? 17. What is the impact of the introduction of the WHO LCG on intrapartum care monitoring (intrapartum fetal surveillance, labour progress monitoring)? | 6. What is the effect of the WHO LCG on the provision of supportive care (e.g., companionship, mobility) and the use of clinical interventions (e.g., cesarean section, augmentation of labour, amniotomy, instrumental delivery, fetal monitoring, cervical dilatation monitoring) during labour and childbirth? |
| Maternal and perinatal outcome | 46. Compare maternal and perinatal outcomes of using WHO LCG versus WHO partograph: a quasi-experimental study 47. To improve maternal (reduction of caesarean section) and perinatal outcome (reduction of IP stillbirths, HIE, 5 min Apgar<7) a care bundle approach in cluster RCT; Standard care VS Care bundle (use of labour care guide, trained constant birth companion, intelligent intermittent auscultation using graphic dopplers & staff trained in NN resuscitation.  48. Does the use of the WHO Labour Care guide result in significant improvement in maternal and newborn outcomes compared to the use of the WHO partograph (or the current standard of care)  49. Do women and neonates who attend high performing LCG sites have improved clinical and psychological outcomes when compared to those using lower-performing LCG sites, or sites where LCG has not been implemented it 50. Amongst women experiencing labour with LCG compared to standard partograph (or otherwise usual care), what is the effect of LCG use on health outcomes related to prolonged duration of labour? 51. Does the use of the labour care guide a) reduce labour interventions, B) reduce maternal and neonatal morbidity/mortality outcomes?  52. Does high-quality implementation of the LCG improve perinatal outcomes such as intrapartum stillbirth, neonatal mortality, near maternal miss? 53. To investigate the effect of the WHO LCG compared to the WHO partograph on maternal and neonatal outcomes and obstetric interventions through a multicentre cluster-randomized strategy trial in high resource settings 54. Does the WHO LCG improve birth outcomes? Method: Large cluster-randomized trial - could be step wedge design (clusters: health facilities) Participants: Health facility staff. Intervention: Workshops with instructions and motivation to implement LCG, ongoing facilitation, and mentoring with on-site champions. Control: Workshops with instructions and motivation to implement the old Partograph, ongoing facilitation, and mentoring with on-site champions. Outcomes: Primary: Maternal: caesarean section. Neonatal: Perinatal mortality. Secondary: Satisfaction with care; breastfeeding; depression; NICU admission; health worker satisfaction 55. What are the long-term outcomes [e.g., Fistula] for women who have had their labours managed using the LCG compared to the modified partograph? | 28. What is the effect of the WHO LCG compared to other labour monitoring tools on maternal and perinatal outcomes? 29. What is the effect of the WHO LCG compared to other labour monitoring tools on long-term outcomes [e.g., Fistula]? 30. Do women and neonates who attend high-performing LCG sites have improved clinical and experience outcomes compared to those attending lower-performing LCG sites (or sites where LCG has not been implemented)? (new) 31. What is the effect of the introduction of WHO LCG on maternal and perinatal outcomes? | 18. What is the impact of the introduction of the WHO LCG on short- and long-term maternal and perinatal morbidity? | 7. What is the effect of the WHO LCG on short- and long-term maternal and perinatal health outcomes? |
| Women experiences | 56. Does the use of the labour care guide improve women's experience with intrapartum care? 57. Impact on the respectful maternity care after introducing WHO LCG: a pre-post intervention study. There are numerous novel validated tools to measure respectful maternity care.  58. Do women who are managed using the WHO Labour Care Guide report a better labour experience compared to women who receive the current standard of care?  59. Do service users who experience labour and birth with the LCG have higher scores in terms of positive labour and birth experiences than those who do not experience labour and birth with the LCG? 60. When a hospital adopts the use of the LCG, does it improve the quality of labour care (measured by women's experience, adherence to WHO guidelines) 61. To determine women's perception and satisfaction on the use of LCG for improved labour outcome 62. Compare childbearing women's experiences when receiving care based on the WHO LCG versus traditional care in countries where the LCG is not implemented. These questions are very important in countries where the WHO LCG is not implemented and share a highly medicalized healthcare system 63. Does high-quality implementation of the LCG improve the qualitative childbirth experience of the parturient?  64. To explore women's experience with birth care using the WHO LCG and the WHO partograph through a multicentre cluster randomized strategy trial in high resource settings | 32. What is the effect of the introduction of WHO LCG on women's satisfaction and experiences of intrapartum care? 33. In different health care settings, does the WHO LCG improve women's satisfaction and experiences of intrapartum care when compared with alternative labour monitoring tools? 34. Do service users who experience labour and birth with the WHO LCG have higher scores in terms of positive labour and birth experiences than those who do not experience labour and birth with the LCG? 35. What is the effect of the introduction of the WHO LCG on respectful maternity (maternal and neonatal) care? | 19. What is the impact of the introduction of the WHO LCG on women's satisfaction and experiences of care during labour and childbirth? 20. What is the impact of the introduction of the WHO LCG on respectful maternity care during labour and childbirth? | 8. What is the effect of the WHO LCG on the experience of care (e.g., satisfaction, respectful maternity care) during labour and childbirth? |
| Tool development | 65. To evaluate the progress of labour in women admitted during the latent phase Population: women admitted during the latent phase of labour (< 5 cm cervical dilatation) Exposure: LCG and co-created clinical guidelines for what to do during the latent phase of labour: see question 1 Design: descriptive study Outcome: duration of labour in latent phase; duration of labour in active phase; timely use of interventions (including artificial rupture of membranes when needed., induction of labour in which phase of labour when needed, oxytocin augmentation (labour progression when started, dose, titration, and duration), AVD during 2nd stage, caesarean section during the latent phase, caesarean section during the active phase of the first stage, caesarean section during the second stage); pain relief during the first stage of labour, perinatal and maternal mortality, cost-effectiveness, women's and health providers' experiences of the intervention 66. What are the optimum time limits per cm cervical dilation for the LCG? Method: Cluster randomized trial - could be step wedge design (clusters: health facilities) Participants: Health facility staff. Intervention: Workshops with instructions and motivation to implement LCG, ongoing facilitation, and mentoring with on-site champions. Control: Workshops with instructions and motivation to implement a modified LCG with shorter time limits (e.g., 80th percentile upper limit from WHO study), ongoing facilitation, and mentoring with on-site champions. Outcomes: Primary: Maternal: caesarean section. Neonatal: Perinatal mortality. Secondary: Satisfaction with care; breastfeeding; depression; NICU admission; health worker satisfaction 67. LCG chart only considers cervical dilatation over time (with some alert thresholds) and fetal head descent (without alert thresholds) to monitor labour progress. For low-risk women in labour, should cervical effacement evaluation be considered another predictor of the normal progression of labour? 68. LCG chart only considers cervical dilatation over time (with some alert thresholds) and fetal head descent (without alert thresholds) to monitor labour progress. Is there any threshold indicating alert zone for fetal head descent? 69. LCG is recommended for skilled health personnel directly providing labour and childbirth care in all settings, is the LCG (with the current alert thresholds) feasible to implement in settings where access to a higher level of care (i.e., c-section, assisted birth) cannot be assured within one hour of exceeding any threshold? Should these thresholds be adapted in terms of the time needed for access to a higher level of care? 70. The literature reviewed to propose the normal ranges (and the alert thresholds for cervical dilatation) considered mainly parity, the status of membranes, and epidural analgesia as the main predictors, without considering interactions between these variables or with other not measured ones. By electronically capturing the information recorded in the LCG, is it possible to create local standards that, at the same time, can be compared with other settings? Is there any other variable important to take into account? What (and how many) interactions can be measured and modeled? Can predictor models be built with this information? Can these models be modified by the events during the latent phase (i.e., early admission, prolonged latent phase)? Is the LCG suitable for labour inductions once active phase starts? | 36. Is the WHO LCG suitable for labour inductions once active phase starts?  37. What is the effect of lower thresholds of WHO LCG cervical dilatation and time limits of the progress of labour (e.g., 80th percentile upper limit) compared to LCG thresholds on intervention rates and maternal and perinatal outcomes at various levels of care (primary, secondary, tertiary) and in different settings (LMIC vs. HIC)?  38. What are the effects of labour ward admission in active phase labour as per the WHO LCG (from 5cm) compared with earlier admission (below 5cm) on maternal and perinatal outcomes?  39. What are the effects of labour ward admission in active phase labour as per the WHO LCG (from 5cm) compared with earlier admission (below 5cm) on latent phase outcomes?  40. Is there any threshold indicating alert zone for fetal head descent? | 21. Is it feasible, safe, and efficacious to use the WHO LCG to monitor labour progress in special situations such as breech presentation, premature labour, and twin pregnancy? 22. Is it feasible, safe, and efficacious to use the WHO LCG to monitor the progress of labour inductions? 23. What is the effect of lower thresholds of WHO LCG cervical dilatation and time limits of the progress of labour (e.g., 80th percentile upper limit) compared to current recommended WHO LCG thresholds (95th percentile upper limit) on maternal and perinatal outcomes in various levels of care and different settings?  24. What is the effect of lower thresholds of WHO LCG cervical dilatation and time limits of the progress of labour (e.g., 80th percentile upper limit) compared to current recommended WHO LCG thresholds (95th percentile upper limit) on the use of clinical interventions in various levels of care and different settings?  25. Is there any efficacious threshold of fetal descent indicating alert zone for fetal head descent? 26. What are the effects of labour ward admission in active phase labour as per the WHO LCG (from 5cm) compared with earlier admission (below 5cm) on maternal and perinatal outcomes? | 9. Is the use of the WHO LCG to monitor labour progress feasible, safe, and effective in improving outcomes in special situations (e.g., induction of labour, breech presentation, premature labour, epidural analgesia, twin pregnancy)? 10. What is the effect of modifying thresholds in the WHO LCG alert line (e.g., lower thresholds of cervical dilatation and time limits of labour progress, the threshold for fetal descent) on maternal and perinatal outcomes, use of clinical interventions, and organization of care, in various levels of care and different settings? |
| Out of scope | 71. Is there an increased risk of infections with episiotomy, and is there a difference in infections between Midline and Mediolateral?  72. Does routine administration of intravenous fluids protect against postpartum hemorrhage? 73. Quantitative and qualitative research on typology of mistreatment and abuse that women face during childbirth. How does the socio-economic status of women determine the extent of abuse and disrespect? 74. Care by Midwives and its impact on decreasing c-sec deliveries-evidence from developing countries 75. Research on implementing the WHO QED framework by countries and adopting the Monitoring Framework -goals, strategic objectives, implementation framework.  76. Impact of COVID on the provision of respectful care during childbirth. for example, women were denied birth companions citing covid protocols 77. At peripheral/rural healthcare facilities in low- and middle-income countries, is the quality of care delivered to pregnant women during labour evidence-based, and does it follow established care standards?  78. How do we support women in labour (particularly in rural areas) to know when they should come to a facility (active labour)? Considerations latent phase admission associated with an unnecessary intervention. 2-year priority  79. How do healthcare professionals support women who arrive at health facilities in latent phase labour? Consideration balance between gatekeeping & unnecessary admission/intervention. 2-year priority 80. How to best monitor fetal well-being in labour in the high middle- and low-income countries  81. How best to treat poor progress in labour in high middle- and low-income countries 82. Who makes decisions around pain relief/management during labour?  83. Does using information obtained by screening fetuses of apparently healthy pregnant women using a continuous wave Doppler apparatus in late pregnancy or early labour reduce intrapartum and early neonatal morbidity and infant mortality? 84. Would a well-designed mapping targeting different organizational levels within a healthcare facility in LMIC, namely administrative, informational services, personnel, identify major health care quality gaps at all levels in the process of delivering care to pregnant women and their newborns during labour and birth?  85. Magnitude of discrimination and subsequent adverse birth outcomes among people of African descent in the first stage of labour and beyond (comprehensive review encompassing low income in high-income settings, e.g., LACRO and US). 86. Is there an outcomes difference when the "support person" in labour is a doula, a family member, a trained assistant, or an untrained but present individual? 87. Are women provided enough autonomy to make an informed decision?  88. Evidence-based interventions around programs that prevent postpartum depression and psychological disorders after the occurrence of stillbirth.  89. Evidence generation around innovative, sustainable approaches for documentation of the first stage of labour in low-income settings (e.g., take advantage of new approaches towards digitalization in the post COVID era). |  |  |  |

**Table S3. Results of focused searches of research efforts**

| **Registry** | **Results** | **WHO LCG matches** |
| --- | --- | --- |
| 1. World Health Organization – International Clinical Trials Registry Platform | 6 | 3 |
| 1. ClinicalTrials.gov | 370 | 0 |
| 1. Health Canada Clinical Trial Database: | 0 |  |
| 1. EU Clinical Trials Register | 0 | - |
| 1. Deutsches Register Klinischer Studien (German Clinical Trials Register) | 4,204 | 0 (no registry from December 2020, date of publication of the WHO LCG) |
| 1. Netherlands Trial Register | 3475 | 0 (no registry from December 2020, date of publication of the WHO LCG) |
| 1. Swiss National Clinical Trials Portal | 14 | 0 |
| 1. ISRCTN | 0 | - |
| 1. ANZCTR | 6 | 0 |
| 1. Chinese Clinical Trial Registry | Not searched* | - |
| 1. India | 3 | 3 |
| 1. Iranian Registry of Clinical Trials | 81 | 0 |
| 1. Japan Primary Registries Network* | Not searched* | - |
| 1. Clinical Research Information Service - Korea | 0 (38 hits under obstetric classification) | - |
| 1. Philippine Health Research Registry | 0 | - |
| 1. Sri Lanka Clinical Trials Registry | 0 | - |
| 1. Thai Clinical Trials Registry | 0 | - |
| 1. Brazilian Clinical Trials Registry | 35 | 0 |
| 1. Public Cuban Registry of Clinical Trials | 2 | 0 |
| 1. Peruvian Registry of Clinical Trials | Integrated in ICTRP. Requires registration | - |
| 1. Pan African Clinical Trials Registry | Inaccessible | - |
| 1. [Tanzania Clinical Trials Registry](http://www.tzctr.or.tz/) | Inaccessible | - |
| WHO LCG: WHO Labour Care Guide  Searches in 22 relevant trial registries listed in the U.S. Department of Health and Human Services (HHS), [Listing of Clinical Trial Registries \| HHS.gov](https://www.hhs.gov/ohrp/international/clinical-trial-registries/index.html), as of 8 November 2021 and last updated on 17 May 2022.  *not searched due to language limitations | | |

**Fig. S4. Profile of respondents to the WHO LCG scoring online survey (phase 2)**

**Professional background**


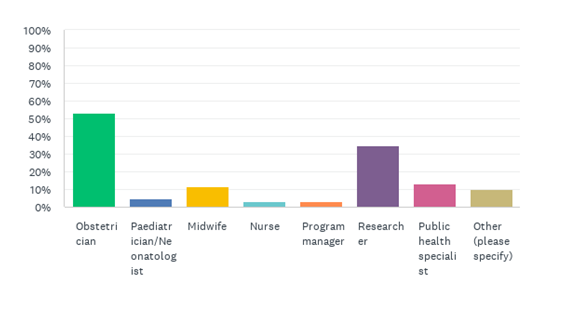

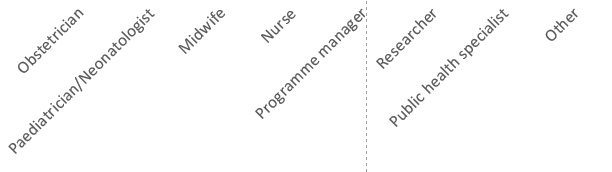


**Main professional affiliation**


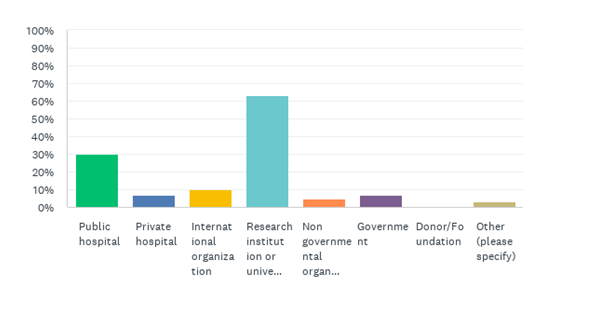

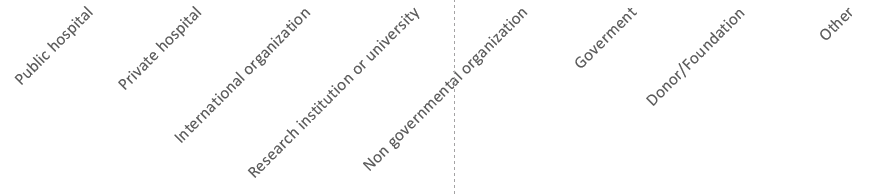


**Region**

**
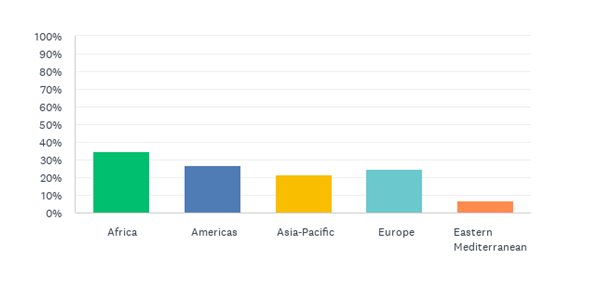
**

**Table S5.A Results of scoring research priorities relating to WHO LCG: total and by domain**

| Rank | Theme | Research Avenue | Average Score* | | | | | | |
| --- | --- | --- | --- | --- | --- | --- | --- | --- | --- |
|  |  |  | Answerability | Effectiveness | Deliverability | Maximum potential impact | Equitability | Timeliness | Total |
| 1 | Implementation research | What are the facilitators and barriers to implementing the WHO LCG with high fidelity among different cadres, levels of care and settings? | 2.80 | 2.55 | 2.69 | 2.48 | 2.64 | 2.64 | 2.63 |
| 2 | Process of care | What is the effect of the WHO LCG on the provision of supportive care (e.g., companionship, mobility) and the use of clinical interventions (e.g., caesarean section, augmentation of labour, amniotomy, instrumental delivery, foetal monitoring, cervical dilatation monitoring) during labour and childbirth? | 2.69 | 2.64 | 2.60 | 2.59 | 2.56 | 2.57 | 2.61 |
| 3 | Women experiences | What is the effect of the WHO LCG on experience of care (e.g., satisfaction, respectful maternity care) during labour and childbirth? | 2.73 | 2.57 | 2.60 | 2.45 | 2.60 | 2.63 | 2.59 |
| 4 | Maternal and perinatal outcome | What is the effect of the WHO LCG on short- and long-term maternal and perinatal health outcomes? | 2.73 | 2.64 | 2.55 | 2.41 | 2.59 | 2.33 | 2.54 |
| 5 | Implementation research | What are the most effective approaches (e.g., education and training, monitoring and feedback, digital LCG, ownership of the WHO LCG and presentation in a health facility by the woman) to implement WHO LCG with high fidelity among different cadres, levels of care and settings? | 2.65 | 2.53 | 2.52 | 2.44 | 2.59 | 2.40 | 2.52 |
| 6 | Implementation research | What are the most effective education and training approaches on the WHO LCG to improve knowledge, attitudes, and intrapartum care practices of different cadres? | 2.63 | 2.44 | 2.55 | 2.48 | 2.39 | 2.44 | 2.49 |
| 7 | Tool development | Is the use of the WHO LCG to monitor labour progress feasible, safe and effective in improving outcomes in special situations (e.g., induction of labour, breech presentation, premature labour, epidural analgesia, twin pregnancy)? | 2.63 | 2.63 | 2.41 | 2.32 | 2.44 | 2.45 | 2.48 |
| 8 | Organization of care and resource utilization | What is the effect of the WHO LCG on the organization of labour and childbirth care (e.g., referrals, teamwork, 5patient flow, human and physical resource requirements, shared decision making between the woman and maternity care provider)? | 2.37 | 2.27 | 2.35 | 2.29 | 2.51 | 2.44 | 2.37 |
| 9 | Tool development | What is the effect of modifying thresholds in the WHO LCG alert line (e.g., lower thresholds of cervical dilatation and time limits of labour progress, threshold for fetal descent) on maternal and perinatal outcomes, use of clinical interventions and organization of care, in various levels of care and different settings? | 2.36 | 2.45 | 2.44 | 2.32 | 2.36 | 2.27 | 2.37 |
| 10 | Organization of care & resource utilization | How cost-effective is the WHO LCG in various levels of care and different settings? | 2.40 | 2.31 | 2.31 | 2.23 | 2.28 | 2.36 | 2.32 |

****Highest possible score – 3.00 (highest priority). Survey results for all respondents n = 75***

**Table S5.B Results of scoring research priorities relating to WHO LCG: all respondents and TWG**

| Theme | Research Avenue | All Participants  n = 75 | | Technical Working Group  n = 13 | |
| --- | --- | --- | --- | --- | --- |
|  |  | Average Score | Rank | Average Score | Rank |
| Implementation research | What are the facilitators and barriers to implementing the WHO LCG with high fidelity among different cadres, levels of care and settings? | 2.63 | 1 | 2.81 | 2 |
| Process of care | What is the effect of the WHO LCG on the provision of supportive care (e.g., companionship, mobility) and the use of clinical interventions (e.g., caesarean section, augmentation of labour, amniotomy, instrumental delivery, foetal monitoring, cervical dilatation monitoring) during labour and childbirth? | 2.61 | 2 | 2.91 | 1 |
| Women experiences | What is the effect of the WHO LCG on experience of care (e.g., satisfaction, respectful maternity care) during labour and childbirth? | 2.59 | 3 | 2.74 | 5 |
| Maternal and perinatal outcome | What is the effect of the WHO LCG on short- and long-term maternal and perinatal health outcomes? | 2.54 | 4 | 2.81 | 3 |
| Implementation research | What are the most effective approaches (e.g., education and training, monitoring and feedback, digital LCG, ownership of the WHO LCG and presentation in a health facility by the woman) to implement WHO LCG with high fidelity among different cadres, levels of care and settings? | 2.52 | 5 | 2.62 | 6 |
| Implementation research | What are the most effective education and training approaches on the WHO LCG to improve knowledge, attitudes, and intrapartum care practices of different cadres? | 2.49 | 6 | 2.78 | 4 |
| Tool development | Is the use of the WHO LCG to monitor labour progress feasible, safe and effective in improving outcomes in special situations (e.g., induction of labour, breech presentation, premature labour, epidural analgesia, twin pregnancy)? | 2.48 | 7 | 2.56 | 7 |
| Organization of care and resource utlization | What is the effect of the WHO LCG on the organization of labour and childbirth care (e.g., referrals, teamwork, patient flow, human and physical resource requirements, shared decision making between the woman and maternity care provider)? | 2.37 | 8 | 2.46 | 9 |
| Tool development | What is the effect of modifying thresholds in the WHO LCG alert line (e.g., lower thresholds of cervical dilatation and time limits of labour progress, threshold for fetal descent) on maternal and perinatal outcomes, use of clinical interventions and organization of care, in various levels of care and different settings? | 2.37 | 9 | 2.47 | 8 |
| Organization of care & resource utilization | How cost-effective is the WHO LCG in various levels of care and different settings? | 2.32 | 10 | 2.31 | 10 |

**Table S6. Rapid mapping of feasibility and acceptability of WHO Labour Care Guide**

The table below summarizes the findings of a rapid review of potential facilitators and barriers for use and implementation of the WHO LCG.

| **Facilitators** | **Barriers** |
| --- | --- |
| - Support implementation of the 2018 WHO intrapartum care recommendations for a positive childbirth experience – mainly around supportive care - Emphasis on positive childbirth experience, woman-centred and supportive care during labour and childbirth - Potential to reduce unnecessary interventions during labour and childbirth - Potential to facilitate quality improvement - Inclusion of section to monitor the woman and baby during second stage of labour - Provides alert values across all observations | - Limited impact of labour tools on maternal and perinatal health outcomes, based on findings from previous partograph - Time required for gradual introduction of the tool - Need for additional training, refresher training and supportive supervision of maternity care providers - Need for women and community education - Too frequent assessments during labour is unrealistic in many settings - Fetal well-being is no longer at the top and could therefore be perceived as less important by maternity providers - Need to develop protocols on management of labour and childbirth complications - Perception that further research of the effect of modified labour progression thresholds on perinatal outcomes is needed - Exclusion of latent first stage of labour - Multiple health systems barriers for labour monitoring and management (e.g. lack of staff in labour ward, lack of medicines and supplies, overcrowding) |

**Table S7. Ranking of research priorities related to WHO LCG: results of the consensus-building process* (phase 3)**

| **Consensus-based ranking** | **Consensus-based theme** | **Consensus-based research avenue** | **Initial ranking**** | **Justification for changes in research avenues** | **Justification for changes in ranking** |
| --- | --- | --- | --- | --- | --- |
| 1 | Optimize implementation strategies of WHO LCG | What are the most effective approaches* to implement WHO LCG with high fidelity among different cadres, levels of care, and settings?  *, e.g., education and training, monitoring and feedback, digital LCG, ownership of the WHO LCG, and presentation in a health facility by the woman | 1,5,6 | This research avenue resulted from merging three questions under the original theme "implementation research". It was considered that the development and testing of any implementation approaches/strategies require an understanding of barriers and facilitators for the introduction and use of WHO LCG. Education and training were also considered key components of any implementation strategy. | There is an urgent need to introduce the WHO LCG, accompanied by effective strategies to maximize compliance with the tool and provide evidence-based intrapartum care practices. This must include studies to improve understanding of successful implementation approaches in different contexts (cadres of skilled personnel, levels of care, and settings)  It is expected that better monitoring of labour and childbirth with WHO LCG will improve process, health, and experience outcomes. The WHO LCG is considered safe for use in most settings and populations.  Any new tool/interventions for implementing WHO LCG in "real-life" needs to be accompanied by rigorous monitoring and evaluation, including consideration to assess unintended consequences (e.g., safety in certain groups). |
| 2 | Improve understanding of the effect of WHO LCG on pregnancy outcomes | What is the effect of the WHO LCG on maternal and perinatal outcomes, and the process and experience of labour and childbirth care*?  *, e.g., the provision of care/use of interventions, the experience of care, short-term maternal and perinatal health, long-term health outcomes | 2,3,4 | This research avenue resulted from merging three research avenues covering the themes "process of care", "women's experiences" and "maternal and perinatal outcome". Process, health and experience outcomes are interrelated and can be investigated under the same or separate research studies. | Generation of robust evidence on the effectiveness of WHO LCG on process, health, and experience outcomes is needed to support adoption, increase acceptability, and use of the tool. The target users may remain sceptical about whether the tool is safe and effective, and this will negatively affect implementation and wider scale-up.  Answering this question to build confidence in the WHO LCG does not impede implementation activities at scale (priority 1). |
| 3 | Assess the effect of the WHO LCG in special situations or particular settings | Is the use of the WHO LCG to monitor labour progress feasible, safe, and effective in improving outcomes in special situations* or particular settings**?  (*, e.g., induction of labour, breech presentation, premature labour, epidural analgesia, twin pregnancy, previous caesarean section)  **(e.g., limited or no access to caesarean section, remote areas, lower-level health facilities) | 7,9 | This research avenue resulted from merging two questions on tool development. Assessment of the effect of the WHO LCG in different populations considered at high risk of developing complications during labour and childbirth or in particular settings, may require similar research designs looking at feasibility, safety, efficacy and effectiveness. | The key populations for WHO LCG use are healthy pregnant women in labour. Therefore, priority should be given to assessing the WHO LCG in its current form before proposing adaptations for special situations or particular settings (e.g., settings without immediate access to caesarean section)  Generation of robust evidence on the feasibility, safety, efficacy, and effectiveness of WHO LCG could inform adaptations of the tool (e.g., modifications of thresholds in the WHO LCG in settings with limited access to caesarean section) and its implementation strategies in special situations or particular settings (e.g. strategies to ensure timely and safe referrals to higher-level facilities or care level, or in special circumstances), including specific protocols for management at different levels of care.  This is particularly relevant in settings with overmedicalized labour care (e.g., high frequency of augmentation, caesarean section), special situations (e.g., inductions of labour, epidural, vaginal births for breech or previous caesarean section) or in settings with limited or no access to caesarean section. Some of these situations may be less common in other settings.  It is worthy to note that having multiple tools for different situations or settings could be harmful for implementation.  Implementing single components of the WHO LCG could also be covered in effectiveness studies looking at the effects of WHO LCG on maternal and perinatal health, experience of care and process outcomes (priority 2). |
| 4 | Understand the effects of WHO LCG on the organization of care and resource requirements | What is the effect of the WHO LCG on the organization of labour and childbirth care* in various levels of care and different settings?  * (e.g., referrals, teamwork, patient flow, human and physical resource requirements, shared decision making between the woman and maternity care provider) | 8 | No changes to the original research avenue. | It was considered more important to generate robust evidence on the effects of WHO LCG on maternal and perinatal health, experience of care and process outcomes than to investigate its effects on organization of care. This research avenue ranked low in the online scoring survey. |
| 5 | Assess the economic impact of the WHO LCG | How cost-effective is the WHO LCG in various levels of care and different settings? | 10 | No changes to the original research avenue. | Estimation of costs and health gains resulting from the use of the WHO LCG (and its implementation strategies) are important to inform healthcare decisions and ensure sustainability.  Cost-effectiveness may be more relevant for the evaluation of the WHO LCG implementation strategy than the tool alone. Economic evaluations can be conducted along with implementation and research efforts; therefore, this question was considered less time critical. Economic evaluations could leverage the information gathered from other studies. This research avenue was ranked last in the online scoring survey. |

*Consensus-building process through virtual meeting including 20 experts; **based on results of the online scoring survey

**Table S8. Ongoing research covering research priorities related to WHO LCG and key milestones**

| **Theme** | **Are there any ongoing or planned research efforts? *** | **Rapid mapping of existing evidence**** | **Key milestones** |
| --- | --- | --- | --- |
| Optimize implementation strategies of WHO LCG | 1. [Labor Care Guide ratings by doctors](http://ctri.nic.in/Clinicaltrials/pdf_generate.php?trialid=67265&EncHid=&modid=&compid=%27,%2767265det%27) (Lead: AG Radhika /Country: India/ Status: completed/ Funder or Sponsor: University College of Medical Sciences, University of Delhi, Guru Teg Bahadur Hospital, Delhi/CTRI/2022/03/041531 )^1^ | WHO LCG usability and feasibility study identified some barriers and facilitators for implementation of WHO^2^  Indirect evidence from partograph:   - There is little evidence to determine the most effective method of partograph implementation. Challenges of partograph implementation are well described (e.g., effective supervision, regular training and refresher training, practical application)^3^ - Monitoring and audit of the partograph in practice, including completion, decision making, referrals and outcomes, has been recommended^3^ | Platform to maximise harmonized data collection across studies, research networks collaboration, and to facilitate evidence synthesis:   - Generic protocols for formative research and co-development of implementation strategies, including data collection tools - Generic monitoring and evaluation tools for national and subnational introduction of WHO LCG and evaluation of the sustainability of WHO LCG.   Generic baseline assessment tools on the provision of facility-based intrapartum care.  Living repository of ongoing WHO LCG introduction and implementation studies, including descriptions of implementation strategies and training materials.  Engagement with women and communities to bring their voices to development of implementation strategies.  Theory of change and conceptual framework for the introduction of WHO LCG.  Interactive Community of Practice (CoP) to share lessons learned. |
| Improve understanding of effect of WHO LCG on outcomes | 1. [Labour Care Guide Trial for reducing Caesarean Section in India](http://www.ctri.nic.in/Clinicaltrials/pmaindet2.php?trialid=50028): Implementing the WHO Labour Care Guide to reduce the use of caesarean section in four hospitals in India: a pragmatic, stepped-wedge, cluster-randomized pilot trial. (Lead(s): Shivaprasad S Goudar, Yeshita V Pujar, J Vogel – Country: India; Status: recruitment completed/ Funder or Sponsor: Bill and Melinda Gates Foundation/ Study registration:).^4^ 2. [To study the effect on caesarean section rate by monitoring the labour progression with the use of 2 different partographs](http://ctri.nic.in/Clinicaltrials/pdf_generate.php?trialid=59594&EncHid=&modid=&compid=%27,%2759594det%27): (Lead: S Agarwal/Country: India/ Status: not yet recruiting/ Funder or Sponsor: AIIMS Jodhpur/ Study registration: CTRI/2021/10/037302)^5^ 3. Effects of introduction of WHO LCG on perinatal outcomes (Lead: C Bedwell/Country: the Zambia and Zimbabwe/ Status: planned/ Funder or Sponsor: not available/ Study registration: not available). 4. Implementation of the WHO Labour Care Guide: a quality improvement project with pre-and post-intervention evaluation (the Better Outcomes for the WHO Labour care guide (BOWL) project) (Lead(s): Badani Moreri-Ntshabele and Justus Hofmeyr/Country: Botswana/ Status: Ongoing/ Funder or Sponsor: not available/ Study registration: not available). 5. Effects of introduction of WHO LCG with early labour support for women in latent phase (Lead: S Downe, V Hundley, T Lavender /Country: England/ Status: planned/ Funder or Sponsor: not available/ Study registration: not available). 6. The Norwegian WHO Labour Care Guide trial: a stepped wedge non-inferiority trial for safety and wellbeing in labour (Lead: S Bernitz /Country: Norway/ Status: planned/ Funder or Sponsor: not available/ Study registration: not available). 7. [Can the use of a next generation partograph based on WHO’s latest intrapartum care recommendations improve neonatal outcomes? A stepped-wedge cluster randomized trial (PICRINO)](https://clinicaltrials.gov/ct2/show/NCT05560802) (Lead: M Blomberg /Country: Sweden/ Status: not yet recruiting/ Funder or Sponsor: Linkoeping University/ Study registration: NCT05560802).^6^ 8. [Impact of WHO Labor Care Guide on reducing cesarean sections at a tertiary center: an open-label randomized controlled trial](https://ctri.nic.in/Clinicaltrials/showallp.php?mid1=57053&EncHid=&userName=CTRI/2021/09/036189) (Lead: D Pandey /Country: India/ Status: published/ Funder or Sponsor: VMMC and Safdarjung Hospital / Study registration: CTRI/2021/09/036189).^7^ | WHO LCG usability, acceptability, and feasibility study: 1200 healthy women, 6 countries, 12 facilities with access to caesarean section, resulted in good birth outcomes and low caesarean section (7%)^2^  Indirect evidence from partograph: evidence from RCTs suggests there is limited effect of use of partograph on improvement in clinical outcomes^3,8,10^ may result in reduction in use of intrapartum interventions^8,10^  Few evaluations of experience or quality of care with no effects found on maternal satisfaction^7^  Indirect evidence from cRCTs: comparison of WHO partograph with Zhang’s guidelines failed to show any differences in intrapartum caesarean section rate in Norway. No maternal or neonatal deaths reported^11,12^ | Platform to facilitate collaboration to implement research studies for evaluation of effectiveness of WHO LCG.  Platform to maximise harmonized data collection across trials and research networks collaboration, and facilitate evidence synthesis:   - Generic protocols for formative research and co-development of implementation strategies, including data collection tools - Core outcomes set for studies evaluating the effectiveness of WHO LCG on maternal and perinatal health, experience of care and process outcomes related to labour and childbirth   Living repository of ongoing studies evaluating effects of WHO LCG. |
| Assess the effect of the WHO LCG in special situations or particular settings | None identified |  | Coordinate collaboration to implement research studies for evaluation of effectiveness of WHO LCG  Living repository of ongoing studies evaluating safety, efficacy, and effectiveness of WHO LCG in special situations or particular settings  Living repository of adaptations of WHO LCG for special situations or particular settings |
| Organization of care and resource utilization | None identified | Usability and feasibility study suggest WHO LCG has the potential to improve communication and team work^2^  Indirect evidence from partograph: partograph is a trigger for referral, but it is unclear if referrals made as a result of partograph use are appropriate; limited effects as a communication tool at handover of care or during referrals ^3^ | Engagement with women and communities to bring their voices to decision-making processes |
| Assess economic impacts of the WHO LCG | None identified | Indirect evidence from partograph: No evidence of evaluation of the cost-effectiveness of the partograph ^3^ | Develop list of relevant cost items for economic evaluations |

**Based on reporting from TWG and* *focused searches (as of 17 May 2022) in major trial registries using key word “Labour Care Guide” and classified based main objectives of the study. Status of identified research efforts last updated on February 13,2023. **Based on published global systematic reviews, realistic reviews published, and large studies published in the last 5 years, as of 08 November 2021.*

References

1. Radhika A. WHO Modified Partogram versus Labour Care Guide (LCG) for Labour Monitoring [Internet]. Clinical Trials Registry - India (CTRI/2022/03/041531). 2022 [cited 2023 February 13]. Available from: http://ctri.nic.in/Clinicaltrials/showallp.php?mid1=67265&EncHid=&userName=CTRI/2022/03/041531

2. Vogel JP, Comrie-Thomson L, Pingray V, Gadama L, Galadanci H, Goudar S, et al. Usability, acceptability, and feasibility of the World Health Organization Labour Care Guide: A mixed-methods, multicountry evaluation. 2021 Mar 1;48(1):66–75.

3. Bedwell C, Levin K, Pett C, Lavender DT. A realist review of the partograph: when and how does it work for labour monitoring? BMC Pregnancy Childbirth. 2017 Dec 13;17(1):31.

4. Vogel JP, Althabe F, Gibbons L, Berrueta M, Pujar Y, Rodriguez R, et al. Implementing the WHO Labour Care Guide to reduce the use of Caesarean section in four hospitals in India: protocol and statistical analysis plan for a pragmatic, stepped-wedge, cluster-randomized pilot trial. Reprod Health. 2023 Jan 20;20(1):18

5. Agarwal S, Singh P. To compare the rate of cesarean section with the use of new WHO partograph and the old WHO partograph - A randomized controlled trial [Internet]. Clinical Trials Registry - India CTRI/2021/10/037302. 2021 [cited 2023 February 13]. Available from: http://ctri.nic.in/Clinicaltrials/showallp.php?mid1=59594&EncHid=&userName=CTRI/2021/10/037302

6. Marie Blomberg. Can the Use of a Next Generation Partograph Improve Neonatal Outcomes? (PICRINO) [Internet].. ClinicalTrials.gov (NCT number): NCT05560802 [cited 2023 February 13]. Available from: https://clinicaltrials.gov/ct2/show/NCT05560802?id=NCT05560802&draw=2&rank=1&load=cart

7. Pandey D, Bharti R, Dabral A, Khanam Z. Impact of WHO Labor Care Guide on reducing cesarean sections at a tertiary center: an open-label randomized controlled trial. AJOG Glob Rep. 2022 Jul 20;2(3)

8. Lavender T, Cuthbert A, Smyth RMD. Effect of partograph use on outcomes for women in spontaneous labour at term and their babies. Cochrane database Syst Rev. 2018 Aug 6;8(8):CD005461.

9. Housseine N, Punt MC, Browne JL, Meguid T, Klipstein-Grobusch K, Kwast BE, et al. Strategies for intrapartum foetal surveillance in low- and middle-income countries: A systematic review. PLoS One. 2018 Oct 1;13(10):e0206295.

10. World Health Organization. Maternal Health and Safe Motherhood Programme. The Partograph: the application of the WHO partograph in the management of labour, report of a WHO multicentre study, 1990-1991. Geneva: World Health Organization; 1994. 251 p.

11. Bernitz S, Dalbye R, Zhang J, Eggebø TM, Frøslie KF, Olsen IC, et al. The frequency of intrapartum caesarean section use with the WHO partograph versus Zhang’s guideline in the Labour Progression Study (LaPS): a multicentre, cluster-randomised controlled trial. Lancet (London, England). 2019 Jan 26;393(10169):340–8.

12. Rozsa DJ, Dalbye R, Bernitz S, Blix E, Dalen I, Braut GS, et al. The effect of Zhang’s guideline vs the WHO partograph on childbirth experience measured by the Childbirth Experience Questionnaire in the Labor Progression Study (LaPS): A cluster randomized trial. Acta Obstet Gynecol Scand. 2022 Feb 1;101(2):193–9.
